# Chemotherapy Negates the Effect of SDF1 mRNA to Distant Metastasis and Poor Overall Survival in Breast Cancer Patients

**DOI:** 10.31557/APJCP.2021.22.3.757

**Published:** 2021-03

**Authors:** Kristanto Yuli Yarso, Monica Bellynda, Akhmad Azmiardi, Brian Wasita, Didik Setyo Heriyanto, Indwiani Astuti, Mohammad Hakimi, Teguh Aryandono

**Affiliations:** 1 *Department of Surgery, Oncology Division, Medical Faculty, Sebelas Maret University, Indonesia. *; 2 *Department of Surgery, Medical Faculty, Sebelas Maret University, Indonesia. *; 3 *Department of Public Health, Faculty of Public Health, Veteran Bangun Nusantara Sukoharjo University, Indonesia. *; 4 *Department of Anatomical Pathology, Medical Faculty, Sebelas Maret University, Indonesia. *; 5 *Department of Anatomical Pathology, Medical Faculty, Gadjah Mada University, Indonesia. *; 6 *Department of Pharmacology and Therapy, Medical Faculty, Gadjah Mada University, Indonesia. *; 7 *Department of Obstetrics and Gynecology, Medical Faculty, Gadjah Mada University, Indonesia. *; 8 *Department of Surgery, Oncology Division, Medical Faculty, Gadjah Mada University, Indonesia. *

**Keywords:** Nuclear CXCR4, cytoplasmic CXCR4, mRNA SDF1a, breast cancer, metastasis, chemotherapy

## Abstract

**Objective:**

Investigate the effect of SDF1a, nuclear, and cytoplasmic CXCR4 breast cancer tissue on metastasis and overall survival in patients with complete-chemotherapy and no-chemotherapy.

**Methods:**

Cohort ambidirectional design was employed with survival analysis that followed the patient’s diagnosis until obtaining the outcome, distant metastasis, or death. We analyzed samples in three groups (all-patient, no-chemotherapy, and complete-chemotherapy groups). Breast cancer cell nuclear and cytoplasm expressions of CXCR4 protein were examined using immunohistochemistry. Amplification of mRNA SDF1a of breast cancer tissue was examined using rtPCR on 131 samples from the same initial paraffin block.

**Results:**

In the distant metastasis and Overall Survival (OS) analysis, there was no correlation between cytoplasmic and nuclear CXCR4 in all-patient, no-chemotherapy, and complete-chemotherapy groups. SDF1a was significantly correlated to shorter distant metastasis and poor OS in the all-patient (p=0.004 and p=0.04, respectively) and no-chemotherapy group (p=0.008 and p=0.026, respectively). However, in the complete-chemotherapy group, SDF1a was not correlated to either metastasis (p=0.527) or OS (p=0.993), advanced stage demonstrated a strong association on shorter distant metastatic in no-chemotherapy (p=0.021) and complete-chemotherapy group (p=0.004) and also poor OS in both groups (p=0.006 and p=0.002, respectively). The hormone receptor showed a protective effect on the no-chemotherapy group’s OS (p= 0.019). Meanwhile, not undergoing chemotherapy was associated with poor OS in the all-patient group (p= 0.011).

**Conclusion:**

SDF1a mRNA amplification has a significant correlation with the occurrence of metastasis and OS in all-patient and no-chemotherapy group. Undergoing chemotherapy negates the effect of SDF1a for distant metastasis and OS.

## Introduction

Breast cancer is a health problem in women around the world. In 2018, it was the second common disease with the fifth-highest mortality worldwide. Total cases of breast cancer were 2,088,849 in 2018. Indonesia ranked first with a total of 58,256 patients (WHO, 2019). In Denpasar, Indonesia, more than 90 new cases of breast cancer are found each year. From the data of 678 patients, more than 43% of breast cancer patients are in local advanced stage III, while 26% have metastases (Yarso et al., 2012).

One crucial component of metastasis is the axis stromal cell-derived factor 1α (SDF1a) with its receptor CXCR4 (Chemokine Motif Receptor 4). Some studies suggest that SDF1a from the cancer microenvironment will spur cancer cells to migrate and associated with metastasis (Kang et al., 2009; Liu et al., 2009). Others stated that SDF1a holds cancer cells in tumor tissue and prevents metastasis (Mirisola et al., 2009).

In a Korean study, high expression of *CXCR4 *on membranes, cytoplasm, and breast cancer cell nuclei was associated with younger age, large tumor size, and poor OS. Another study has shown that nuclear *CXCR4* expression and lymph node metastases are associated with the prognosis of breast cancer patients. Several studies have shown that *CXCR4* expression in the membranes is associated with prognostic factors, but nuclear *CXCR4* expression is not (Woo et al., 2008; Kim et al., 2009; Yasuoka et al., 2008). CXCR4 cycle after exposure to high SDF1a results in endocytosis and is in the cytoplasm through an internalization process. This CXCR4 will then be ubiquitinated (Bushillo et al., 2010). Some of these clinical studies show the effects of SDF1a and CXCR4 are not the same and are inconstant. In this study, we investigate chemotherapy as a factor influencing the effect of SDF1a and CXCR4 on distant metastasis and OS. 

## Materials and Methods

*Patients and tissue samples*

The data were obtained from dr. Moewardi and Kasih Ibu Hospital in Surakarta, Central Java, Indonesia. The patients’ data were collected from January 2013 to January 2015. The inclusion criteria are (a) patients with stage 1-3 breast cancer at the time of diagnosis who visited the oncology clinic, (b) patients who could be tracked and followed up via direct or phone interview, and c) patients with accessible paraffin blocks. We observed 131 subjects. Metastasis events were recorded from the diagnosis until they showed symptoms and had distant metastases or deceased. Observations were conducted prospectively until January 2020 for 1-135 months with a median follow-up of 27 months. The examination or tracing of metastasis was performed on schedule or if there was any symptom of metastasis. The subjects who did not show up for examination were examined at home. Observation and recording of the signs of metastasis and death were undergone both in person and through phone interviews. All data were taken from medical records on the baseline and follow-up time.

According to ASCO, immunohistochemistry (IHC) examination with negative ER PR is if the number of stained nuclei in tumor cells is less than 1%, and Her2 negative is if the number of stained nuclei is two or less with negative FISH. The appropriate cut-off point for Ki67 was determined negative if the value is less than 20% (Arima et al., 2019). There were 68 subjects (51.9%) in the complete-chemotherapy group and 63 subjects in the no-chemotherapy group (49.1%). The no-chemotherapy group consists of patients who refused chemotherapy, including patients dropping out of chemotherapy before four sessions for any reason. The chemotherapy regimen used was anthracycline base.

All patients underwent modified mastectomy surgery if possible. Radiation data cannot be analyzed because many patients did not receive the treatment or the therapy was out of the specified schedule for some reason.

The degree of differentiation is determined based on the Scarff-Bloom-Richardson system and classified into three grades (Fitzgibbons et al., 2000). Follow-up was assessed from the time of diagnosis to distant metastases and decease. OS, distant metastases, and locoregional recurrence were calculated in months, determined with clinical, pathological, and radiological data. 

*Rt PCR and IHC*

Hematoxylin-eosin slides from the diagnosis were reviewed. The part containing the tumor was resected by 4µ and examined according to protocol with the primary SDF-1 prepared before. The rt-PCR examination for SDF1 was performed on paraffin blocks from biopsy results. The paraffin blocks were stored at room temperature with age <5 years. To ensure the quality of the mRNA, we did concentration checking before further execution. We used spin column-based nucleic acid purification as the mRNA extraction method. The rt-PCR SDF1a examination results were classified into two groups, high and low, based on median values. 

The mRNA samples were tested using the NEXproTM qRT-PCR Master Mix (SYBR) kit following the manufacturer’s instructions. Quantitative PCR was carried out using Bioneer ExicyclerTM 96 Real-Time Quantitative Thermal Block. The PCR condition was as recommended by the manufacturer. The PCR primers were as follows: forward 5’-CAGAGCCAACGTCAAGCA-3′ and reverse 5′-AGGTACTCTTGGATCCAC-3.

**Figure 1:**
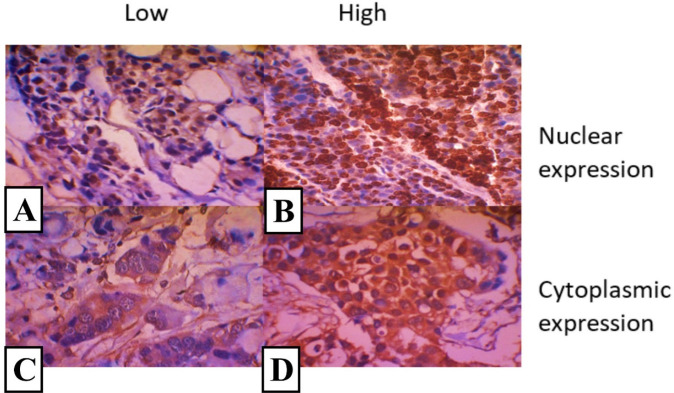
(a). Low nuclear expression, (b). High nuclear expression, (c). Low cytoplasmic expression, (d) High cytoplasmic expression

For CXCR4 examination, an IHC paraffin block was cut by 4µ and stained according to the CXCR4 protocol. We used antibodies from R and D Human CXCR4 Antibody Monoclonal Mouse IgG2B Clone # 44716 Catalog Number: MAB172. The test was performed from the same paraffin block as the SDF1 mRNA sample. The *CXCR4* test was analyzed using two methods: the expression on the nucleus the cytoplasm. The assessment performed using scores calculated with the Image J program was divided into high and low expressions groups (Figure 1). 

Digital images were captured using an Olympus CX21 microscope (Olympus Corporation, Tokyo, Japan) equipped with 10×, 20×, and 40× objective lenses and an Optilab digital color camera (Miconos, Yogyakarta, Indonesia). The images were stored using an uncompressed image file format (bitmap). For every imaging session, an image from an empty slide background area was acquired (blank field image) to correct image color balance and uneven illumination. *CXCR4* expression was analyzed using Image J Software with IHC profiler plugin according to Varghese et al. (2014) and score as negative (0), low positive (1+), positive (2+), and high positive (3+). Score 0-2 were categorized as low expression, and score 3 was categorized as high expression.

*Statistical Analysis*

Patients were followed up and documented from the time of diagnosis until distant metastasis or death. The survival and distant metastasis time of the high and low expression groups was determined using Kaplan-Meier survival analysis , and the characteristic data of both groups were analyzed using the chi-square Fischer exact test when applicable. The survival chart is presented in log-rank. Cox proportional hazards model and logistic regression analysis were used to evaluate the prognostic significance of each variable in the univariate and multivariate analyses. The analysis was conducted on all-patient group followed by no-chemotherapy and chemotherapy groups.

All statistical analyses were performed using SPSS Statistics software version 22.0 (IBM Corp., Armonk, NY, USA). It is statistically significant if the result is less than 0.05. 

## Results

From the histological examination, the pathological diagnosis was invasive ductal carcinoma (IDC) in 116 patients (88.4%), invasive lobular carcinoma (ILC) in 3 patients (2.3%), medullary in 5 patients (3.9%), mucinous in 1 patient (0.8%), papillary in 3 patients (2.3%), and scirrhous in 3 patients (2.3%). The subject’s clinicopathological characteristic is described more in Table 1.

*Expression of CXCR4 and SDF1a*

There was no *CXCR4* expression on cell membranes. High expression of nuclear* CXCR4* was noted in 91 subjects, but 37 others had low expression. There were 17 positive and 111 negative cytoplasmic *CXCR4* expressions. Three IHC samples could not be read. The value of SDF1a amplification ranged from 1 to 11942 with a median of 34. We found 67 samples with high and 64 with low SDF1a amplification. 

There was a correlation between high nuclear CXCR4 and advanced stage, between high cytoplasmic CXCR4 and positive KI67 in bivariate analysis. More than half (51,5%) of the subjects had distant metastasis to the lung, bone, liver, contralateral breast, brain, and others by 25.2%, 8.4%, 5.3%, 4.6%, 2.3%, and 3.1%, respectively. Local recurrence had been noted on 7.6% of the subjects. There were 68 (51.9%) subjects who completed chemotherapy, while 63 (48.1%) of them dropped out of or did not undergo chemotherapy.

*Distant Metastasis*

**Figure 2:**
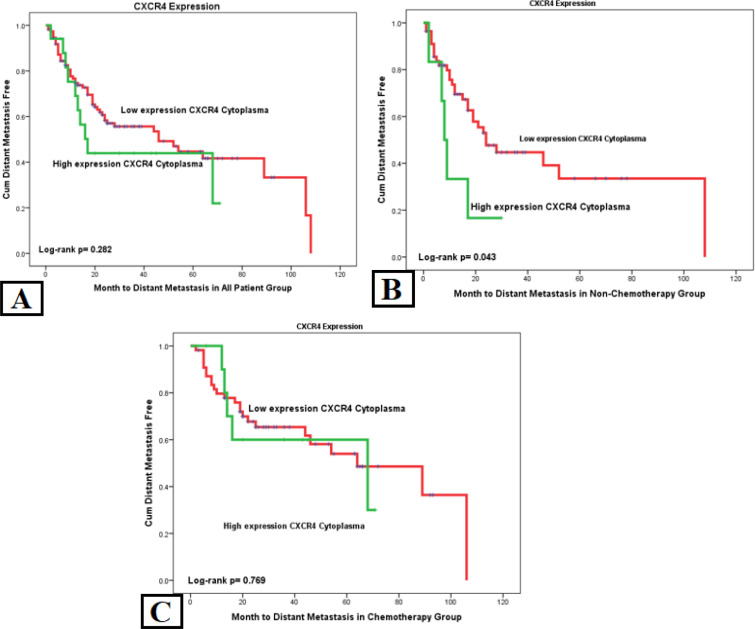
Time from Diagnosis to Distant Metastasis Determined by Cytoplasmic CXCR4. (a) In the all-patient group, the cytoplasmic CXCR4 expression showed a non-significant to correlation to metastasis. (b) However, the non-chemotherapy group showed a significant difference in high cytoplasmic CXCR4 expression with a worse metastasis. (c) Meanwhile, in the complete-chemotherapy group, there was no difference in high and low cytoplasmic CXCR4

**Figure 3:**
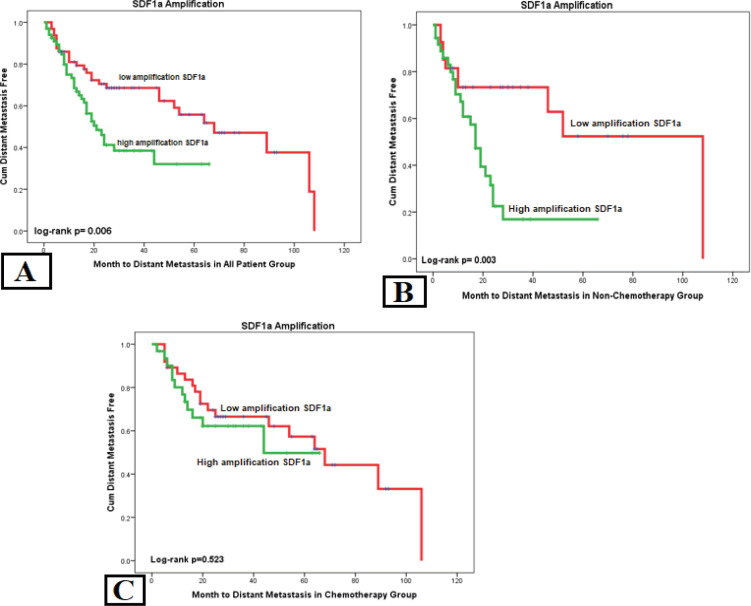
Time from Diagnosis to Distant Metastasis Determined by SDF1a. (a) In the all-patient group, SDF1a high amplification showed a significantly shorter metastasis time. (b) Likewise, the non-chemotherapy group showed a significant difference. (c) Interestingly in the complete-chemotherapy group, there was no difference in high and low SDF1a amplification toward to metastasis

We used the Kaplan-Meier survival curve for distant metastasis based on each marker in the all-patient group. There was a significant difference found in the SDF1a examination of metastasis with log-rank p= 0.004 HR= 1.903. Another significant variable was stage with p= 0.000 HR= 3.966 (Table 2). It means high SDF1 and advanced stage was associated with shorter time of distant metastasis. High cytoplasmic *CXCR4* expression and SDF1a amplification was also associated with shorter time to distant metastasis in no-chemotherapy group (Figures 2 and 3). 

To ensure the effect of chemotherapy on the results of this study, we conducted an analysis of samples divided into two groups, no-chemotherapy and complete-chemotherapy groups. Results showed that SDF1a has a strong correlation in the no-chemotherapy group with p= 0.008 HR= 3.039 but had no correlation in the complete-chemotherapy group. Another significant variable in the no-chemotherapy group is the stage with p= 0.021 HR= 3.534. There were no significant variables for the complete-chemotherapy group, except for stage with p= 0.004 HR= 3.707, which is a constant variable affecting distant metastasis (Table 2).

*Overall Survival*

Analysis of SDF1a with OS in the all-patient group showed a correlation with p=0.040. Another significant variable was chemotherapy with p= 0.011 HR= 2.168 and stage with p= 0.002 HR= 3.870. We analyzed two groups of chemotherapy to eliminate the confounding effect. In the no-chemotherapy group, SDF1a showed a correlation with p=0.026 HR=2.738 and stage with p= 0.006 HR= 6.203. Another variable, the hormone receptor, showed a protective effect on OS with p= 0.019 HR= 0.354. The complete-chemotherapy group showed that only stage was correlated with OS with p= 0.020 HR= 3.823. There was no correlation of SDF1a towards OS in the complete-chemotherapy group (Table 3).

**Figure 4:**
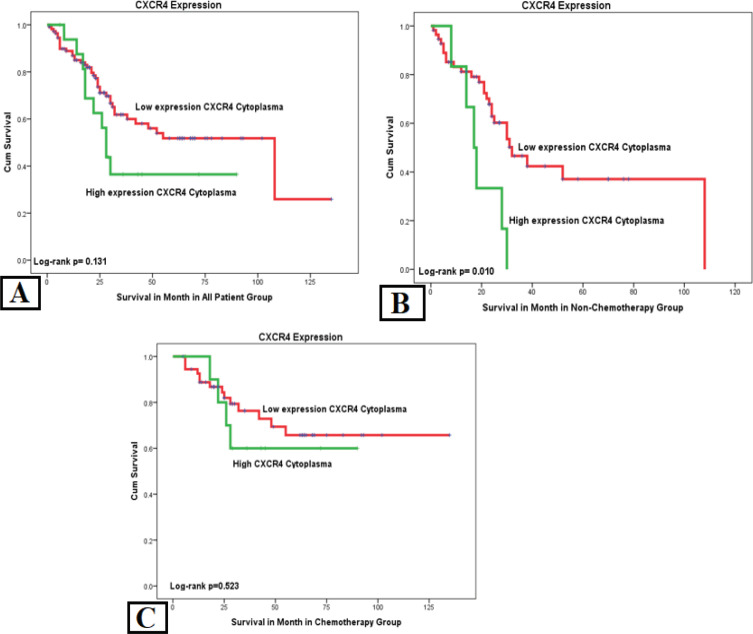
Kaplan–Meier Chart of Cytoplasmic CXCR4 to Overall Survival in All 3 Patient Groups. (a) High cytoplasmic CXCR4 expression showed shorter metastatic time although it was not significant in the all-patient group. (b) High cytoplasmic CXCR4 expression in the no chemotherapy group showed a significant difference in the poor OS with P = 0.010. (c) But interestingly complete-chemotherapy group showed no significant difference between high and low cytoplasmic CXCR4 expressions

**Figure 5:**
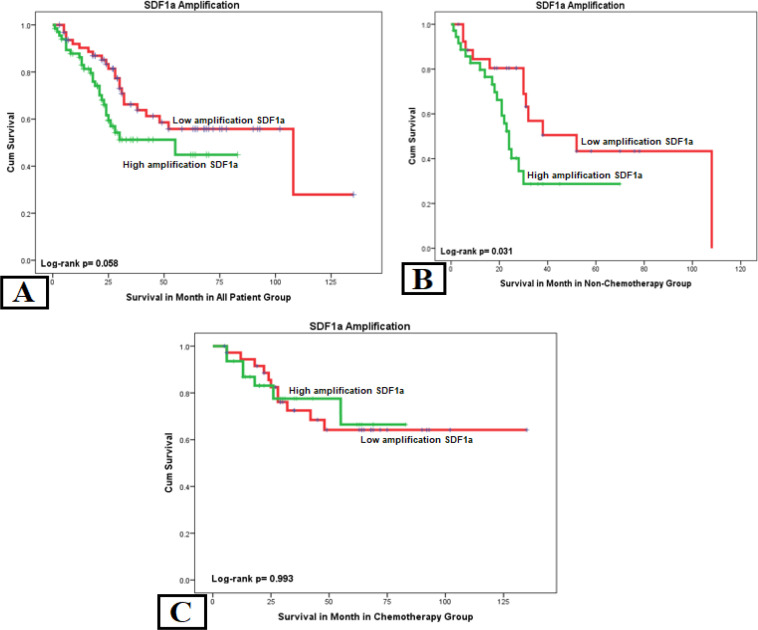
Kaplan–Meier Chart of SDF1a to Overall Survival in All 3 Patient Groups. (a) The SDF1 amplification showed a no significant OS in the all-patient group. (b) High expression of SDF 1 in no-chemotherapy group had significantly different OS. (c) Complete-chemotherapy group showed no significant difference between high and low SDF1a

Stage has the strongest correlation in all-patient, no-chemotherapy, and chemotherapy groups, both on the distant metastasis and the OS. In Kaplan-Meier curve analysis, we found that higher expression of cytoplasmic *CXCR4* and *SDF1a* expression was related with poor OS in the no-chemotherapy group (Figure 4 and 5). Patients with high SDF1a amplification have a shorter mean OS of 25.49 months than low SDF1a amplification with a mean OS of 41.39 months and shorter time to distant metastasis of 19.96 months compared to low SDF1a amplification with a mean of 35.98 months (Table 4). 

**Table 1:** Clinicopathological Characteristics in Patients According to the Expression of *SDF1a, *Cytoplasmic* CXCR4 *and nuclear *CXCR4*

Variable	SDF1a				Cytoplasmic CXCR 4				Nuclear CXCR4			
	N	Low	High	P	N	Low	High	P	N	Low	High	P
Age												
<50	64 (49%)	30 (47%)	34 (53%)	0.73	63 (49%)	57 (90%)	6 (10%)	0.30	63 (49%)	17 (27%)	46 (73%)	0.70
>50	67 (51%)	34 (51%)	33 (49%)		65 (51%)	54 (83%)	11 (17%)		65 (51%)	20 (31%)	45 (69%)	
Stage												
I, II	44 (34%)	24 (55%)	20 (45%)	0.36	43 (34%)	39 (91%)	4 (9%)	0.42	43 (34%)	8 (19%)	35 (81%)	0.01
III	87 (66%)	40 (46%)	47 (54%)		85 (66%)	72 (85%)	13 (15%)		85 (66%)	29 (34%)	56 (66%)	
Chemotherapy												
Complete	68 (52%)	37 (54%)	31 (46%)	0.22	66 (52%)	55 (83%)	11 (17%)	0.30	66 (52%)	21 (32%)	45 (68%)	0.56
Incomplete	63 (48%)	27 (43%)	36 (57%)		62 (48%)	56 (90%)	6 (10%)		62 (48%)	16 (26%)	46 (74%)	
Hormone Status												
Negative	83 (65%)	37 (45%)	46 (55%)	0.19	81 (64%)	69 (85%)	12 (15%)	0.41	81 (64%)	24 (30%)	57 (70%)	0.84
Positive	45 (35%)	26 (58%)	19 (42%)		45 (36%)	41 (91%)	4 (9%)		45 (36%)	12 (27%)	33 (73%)	
HER-2												
Negative	70 (55%)	33 (47%)	37 (53%)	0.72	70 (56%)	63 (90%)	7 (10%)	0.42	70 (56%)	18 (26%)	52 (74%)	0.44
Positive	58 (45%)	30 (52%)	28 (48%)		56 (44%)	47 (84%)	9 (16%)		56 (44%)	18 (32%)	38 (68%)	
KI67												
Negative	48 (37%)	20 (42%)	28 (58%)	0.27	45 (36%)	43 (96%)	2 (4%)	0.05	45 (36%)	15 (33%)	30 (67%)	0.41
Positive	81 (63%)	43 (53%)	38 (47%)		81 (64%)	67 (83%)	14 (17%)		81 (64%)	21 (26%)	60 (74%)	
Grade												
I	2 (2%)	2 (100%)	0 (0%)	0.35	2 (2%)	2 (100%)	0 (0%)	0.54	2 (2%)	0 (0%)	2 (100%)	0.28
II	35 (27%)	18 (51%)	17 (49%)		34 (27%)	31 (91%)	3 (9%)		34 (27%)	13 (38%)	21 (62%)	
III	91 (71%)	44 (48%)	47 (52%)		91 (72%)	77 (85%)	14 (15%)		91 (72%)	24 (26%)	67 (74%)	

**Table 2:** Cox Regression Analysis of Distant Metastases in All-Patient, no-Chemotherapy, and Complete-Chemotherapy Group

Variable	All-Patient Group				No-Chemotherapy Group				Complete-Chemotherapy Group			
	Univariate		Multivariate		Univariate		Multivariate		Univariate		Multivariate	
	HR (CI 95%)	P	HR (CI 95%)	P	HR (CI 95%)	P	HR	P	HR (CI 95%)	P	HR (CI 95%)	P
Age	0.71 (0.43-1.17)	0.182	-	-	0.79 (0.39-1.56)	0.496	-	-	0.58 (0.27-1.27)	0.168	-	-
High SDF1a	2.22 (0.43-1.17)	0.004	1.90 (0.43-1.17)	0.004	3.09 (1.39-6.86)	0.006	3.04 (1.33-6.93)	0.008	1.28 (0.59-2.78)	0.527	-	-
High Nuclear CXCR 4	0.99 (0.43-1.17)	0.979	-	-	0.74 (0.35-1.57)	0.434	-	-	1.24 (0.54-2.83)	0.615	-	-
High Cytoplasmic CXCR4	1.42 (0.72-2.80)	0.312	-	-	2.58 (0.98-6.76)	0.054	1.85 (0.69-4.96)	0.218	1.16 (0.44-3.06)	0.771	-	-
KI67 Positive	1.12 (0.66-1.89)	0.670	-	-	1.36 (0.63-2.95)	0.432	-	-	0.83 (0.39-1.75)	0.630	-	-
High Grade	1.79 (0.99-3.20)	0.051	1.12 (0.61-2.16)	0.678	2.00 (0.84-4.78)	0.119	-	-	1.46 (0.65-3.25)	0.355	-	-
HER- Positive	0.69 (0.41-1.17)	0.175	-	-	0.74 (0.35-1.57)	0.432	-	-	0.74 (0.35-1.57)	0.438	-	-
Hormone Positive	0.92 (0.55-1.53)	0.747	-	-	0,75 (0.56-1.54)	0.436	-	-	1.19 (0.56-2.64)	0.641	-	-
No Chemotherapy	1.64 (0.99-2.69)	0.053	1.45 (0.87-2.42)	0.157	-	-	-	-	-	-	-	-
Stage	3.99 (2.05-7.75)	0.000	3.97 (1.90-8.28)	0.000	4.19 (1.46-12.01)	0.008	3.53 (1.21-10.35)	0.021	3.62 (1.51-8.65)	0.004	-	-

**Table 3:** Cox Regression Analysis of Overall Survival in All-Patient, no-Chemotherapy, and Complete-Chemotherapy Group

Variable	All-Patient Group				No-Chemotherapy Group				Complete-Chemotherapy Group			
	Univariate		Multivariate		Univariate		Multivariate		Univariate		Multivariate	
	HR (CI 95%)	P	HR (CI 95%)	P	HR (CI 95%)	P	HR (CI 95%)	P	HR (CI 95%)	P	HR (CI 95%)	P
Age	0.82 (0.47-1.43)	0.491	-	-	0.83 (0.41-1.67)	0.599	-	-	0.56 (0.21-1.49)	0.245	-	-
High SDF1a	1.77 (0.99-3.14)	0.051	1.90 (1.03-3.50)	0.040	2.26 (1.05-4.83)	0.036	2.74 (1.13-6.63)	0.026	0.99 (0.38-2.58)	0.993	-	-
High Nuclear CXCR 4	0.72 (0.40-1.29)	0.270	-	-	0.84 (0.39-1.79)	0.650	-	-	0.71 (0.27-1.82)	0.472	-	-
High Cytoplasmic CXCR4	1.68 (0.84-3.37)	0.143	-	-	3.09 (1.24-7.72)	0.016	1.50 (0.49-4.75)	0.478	1.43 (0.47-4.37)	0.528	-	-
KI67 Positive	1.12 (0.62-2.02)	0.704	-	-	2.17 (0.93-5.07)	0.074	1.72 (0.73-4.07)	0.217	0.55 (0.22-1.38)	0.202	-	-
High Grade	2.39 (1.18-4.85)	0.015	1.47 (0.69-3.12)	0.316	3.07 (1.09-8.67)	0.034	2.45 (0.76-7.97)	0.135	2.07 (0.70-6.13)	0.188	-	-
HER-2 Positive	0.59 (0.33-1.08)	0.089	0.67 (0.36-1.22)	0.186	0.63 (0.28-1.43)	0.273	-	-	0.79 (0.32-2.01)	0.634	-	-
Hormone Positive	0.74 (0.44-1.34)	0.323	-	-	0.42 (0.19-0.93)	0.032	0.35 (0.15-0.84)	0.019	1.10 (0.43-2.85)	0.837	-	-
No Chemotherapy	2.72 (1.52-4.88)	0.001	2.17 (1.19-3.94)	0.011	-	-	-	-	-	-	-	-
Stage	5.19 (2.31-11.64)	0.000	3.87 (1.65-9.06)	0.002	6.29 (1.89-20.91)	0.003	6.20 (1.67-23.01)	0.006	3.82 (1.24-11.82)	0.020	-	-

**Table 4:** The Duration to Distant Metastasis and Survival (in Months) Difference between High and Low Amplification of SDF1a, and Expression of Cytoplasm and NuAclear

Variables		n	Mean	SD	p
SDF1a	Month to distant metastasis				
	Low	64 (49%)	35.98	28.39	<0.001
	High	67 (51%)	19.96	16.28	
	Survival in month				
	Low	64 (49%)	41.39	29.25	<0.001
	High	67 (51%)	25.49	18.46	
CXCR4 Cytoplasma	Month to distant metastasis				
	Low	117 (87%)	28.50	25.05	0.537
	High	17 (13%)	24.53	21.20	
	Survival in month				
	Low	117 (87%)	33.61	26.44	0.719
	High	17 (13%)	31.18	21.84	
CXCR4 Nuclear	Month to distant metastasis				
	Low	37 (30%)	28.35	23.23	0.831
	High	87 (70%)	27.32	25.12	
	Survival in month				
	Low	37 (30%)	32.08	21.80	0.772
	High	87 (70%)	33.56	27.51	

## Discussion

In our previous study, the expression of *SDF1a *with *IHC* was higher in groups with distant metastasis. Expressions include cancer cells as autocrine and other cells in the tumor microenvironment as paracrine (Yarso et al., 2016). This process can affect through two mechanisms: first, by direct stimulation of SDF1a to cancer cells that will induce growth and motility to avoid apoptosis and, second, by influencing the microenvironment, among them, by attracting EPC that induces angiogenesis so that cancer cells can intravasate more easily (Asri et al., 2016).

In this study, SDF1a correlated with worse in distant metastases and OS in all-patient and no-chemotherapy groups, but not in the chemotherapy group. Cytoplasmic and nuclear CXCR4 did not have any correlation between metastasis and OS in all-patient, no-chemotherapy, and chemotherapy group. The hormone receptor has a protective effect on OS in the no-chemotherapy group even though advanced stage is continually showing a significant correlation with metastasis and OS in all-patient, no-chemotherapy, and chemotherapy group.

In developing countries like Indonesia, trust in alternative medicine, fear of hair loss, and wrong myths about chemotherapy cause not all patients to undergo or drop out of chemotherapy. Refusing standard chemotherapy alone will reduce survival by more than 30%. Verkooijen (2005) found a decrease of 36% vs. 75% in 10 years. Meanwhile, Joseph (2012) found poor outcomes compared to patients receiving standard chemotherapy by 43.2% vs. 81.9%. From a previous study, administering GSCMF or cyclophosphamide reported decreased SDF-1 concentrations in vivo in rat bone marrow and decreased the accumulation of serine protease, which can directly cleave and regulate SDF-1 proteolytic degradation of SDF-1, along with CXCR4 (Levesque et al., 2003). Unfortunately, this research did not lead to clinical study. This study may answer why patients with high SDF1a in the complete-chemotherapy group are not associated with OS and metastases. In other breast cancer studies with dense chemotherapy with GSCMF, it is believed that dose-dense treatment may partly reflect the inhibition of micrometastasis homing and/or paracrine survival associated with CXCR4 (Epstein, 2004).

Previous research stated that the expression of the *SDF1* score from IHC might be higher in subjects with metastasis. Other research also shows IHC from SDF1 is associated with poor survival and local recurrence, similar to the study of transcription of frozen section tissue using IHC and RtPCR (Kang et al., 2005). In contrast to Italy, Mirisola et al., (2009) stated SDF1a with microarrays and IHC was associated with good DFS and OS. Besides, SDF1 in cancerous tissue can also induce aggressiveness by calling on EPC or modification of the immune system by recruiting lymphocytes (Petit et al., 2007).

The secretion of CXCL12 by breast cancer cells can enhance invasion, recruit macrophages, and increase microvessel density, which may also be mediated by tumor-associated macrophages and contributes to altered tumor architecture. These results demonstrate how a tumor can increase invasion and motility and contribute to enhanced tumor malignancy (Boimel et al., 2012).

Preliminary study in Indonesia about *CXCR4* with *IHC*, the expression is the same for breast cancer with or without distant metastasis, but the high expression of *CXCR4* tends to have metastases to the lungs other organs (Hariyanto, 2012). In China, high nuclear *CXCR4* was associated with negative lymph node metastases, while high cytoplasmic *CXCR4* expression was associated with patients with lymph node metastases (Su et al., 2006). These results contradict the previous study in Korea, which found that high *CXCR4* expression in the cytoplasm was associated with better RFS and OS in triple-negative breast cancer with chemotherapy (Shim et al., 2018).

Theoretically, the high CXCR4 outcomes have more aggressive metastases both in-vitro and in-vivo (Hernandez et al., 2011). In CXCR4 receptors, there are still proteins that affect the sensitivity of the stimulation from SDF1, including GRK and Arestin 2 proteins, influencing the active cascade under CXCR4. Another study has shown a decrease in the sensitivity of CXCR4 to induction from SDF1 by performing endocytosis and degradation (Bushillo et al., 2010).

In the study using stromal mesenchymal cells from the fetus, CXCR4 was only found in the membrane by 4%. The rest in the endosome or lysosome might be at the stage of CXCR4 recycling. There was also CXCR4 in the cell nucleus. The treatment that increases the expression of *CXCR4* in the membrane turns out to increase metastasis 2.6 times (Pelekanos et al., 2014).

In conclusion, SDF1a mRNA amplification has a significant correlation with the occurrence of metastasis and OS in all-patient and no-chemotherapy group. Undergoing chemotherapy negates the effect of SDF1a for distant metastasis and OS. 

## Author Contribution Statement

Kristanto Yuli Yarso: funding, data collection, statistic, primary investigation, supervision, writing; Monica Bellynda: data collection, manuscript, software, writing; Akhmad Azmiardi: format analysis, software; Brian Wasita: data collection (histopathological), data curation; Didik Setyo Heriyanto: data curation, rtPCR; Indwiani Astuti, Mohammad Hakimi: methodology; Teguh Aryandono: conceptualization.
